# Utility of Repeat Sampling in Bilateral Aldosterone Suppression During Adrenal Vein Sampling for Primary Aldosteronism

**DOI:** 10.1210/jcemcr/luae051

**Published:** 2024-04-10

**Authors:** Bella Halim, Eric X Z Yong, Matthew Egan, Richard J MacIsaac, David O’Neal, Nirupa Sachithanandan

**Affiliations:** Department of Endocrinology & Diabetes, St Vincent's Hospital Melbourne, Fitzroy, Victoria 3065, Australia; Department of Medicine, The University of Melbourne, Fitzroy, Victoria 3065, Australia; Department of Radiology, St Vincent's Hospital Melbourne, Fitzroy, Victoria 3065, Australia; Department of Cancer Imaging, Peter MacCallum Cancer Centre, Parkville, Victoria 3000, Australia; Department of Pathology, St Vincent's Hospital Melbourne, Fitzroy, Victoria 3065, Australia; Department of Endocrinology & Diabetes, St Vincent's Hospital Melbourne, Fitzroy, Victoria 3065, Australia; Department of Medicine, The University of Melbourne, Fitzroy, Victoria 3065, Australia; Department of Endocrinology & Diabetes, St Vincent's Hospital Melbourne, Fitzroy, Victoria 3065, Australia; Department of Medicine, The University of Melbourne, Fitzroy, Victoria 3065, Australia; Department of Endocrinology & Diabetes, St Vincent's Hospital Melbourne, Fitzroy, Victoria 3065, Australia; Department of Medicine, The University of Melbourne, Fitzroy, Victoria 3065, Australia

**Keywords:** primary aldosteronism, adrenal vein sampling, apparent bilateral aldosterone suppression, double-down AVS

## Abstract

Primary aldosteronism (PA) is the most common form of secondary hypertension. Accurate subtyping of PA is essential to identify unilateral disease, as adrenalectomy improves outcomes. Subtyping PA requires adrenal vein sampling (AVS), which is technically challenging and results from AVS may not always be conclusive. We present a case of a 37-year-old man with PA whose AVS studies were inconclusive due to apparent bilateral aldosterone suppression (ABAS). As a result, our patient was misdiagnosed as having bilateral PA and medically managed until a repeat AVS showed lateralization to the right adrenal gland. ABAS is an underrecognized phenomenon that may confound the subtyping of PA. We recommend repeating AVS in such cases and discuss strategies to minimize ABAS.

## Introduction

Primary aldosteronism (PA), the most common form of secondary hypertension, is associated with increased cardiovascular morbidity and mortality, and optimal therapy with surgery or mineralocorticoid receptor antagonist is required to improve outcomes (1). Excess aldosterone in PA can be unilateral, caused by an aldosterone-producing adenoma (APA), or bilateral, caused by bilateral adrenal hyperplasia. Rare causes of PA include adrenal carcinoma, familial hyperaldosteronism, and ectopic aldosterone-producing tumors. The diagnostic process in PA can be simplified into 3 distinct steps: case finding, confirmatory, and subtyping tests. Accurate subtyping of PA as unilateral or bilateral is essential, as adrenalectomy for unilateral PA improves clinical outcomes (1, 2) and quality of life (3, 4). Adrenal vein sampling (AVS) is the gold-standard test in subtyping PA; however, its interpretation can be challenging. We describe a case of apparent bilateral aldosterone suppression (ABAS) during AVS, an underrecognized phenomenon and provide strategies for its management.

## Case Presentation

**Figure 1.**
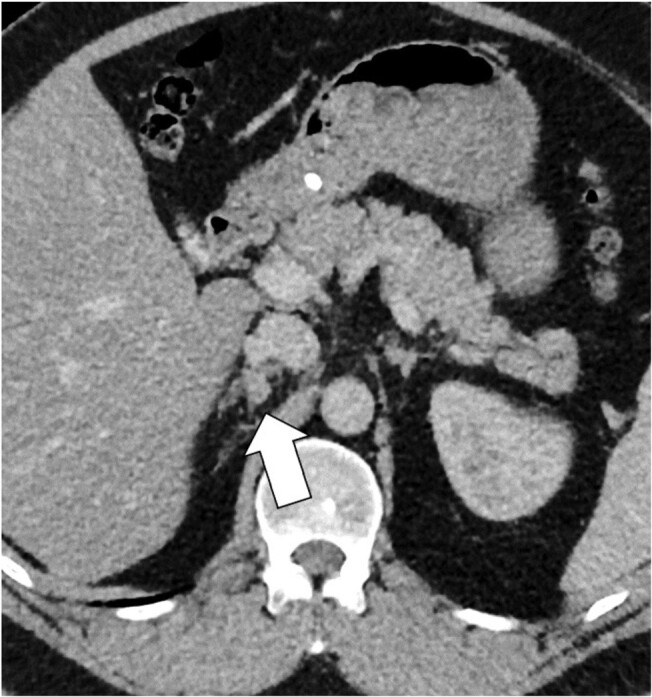
Noncontrast computed tomography image of the adrenal glands. Arrow indicates nodular thickening of the right medial limb.

A 37-year-old man was referred to our clinic for investigation of PA. He presented with hypertension and hypokalemia at 2.6 mmol/L (reference range [RR], 3.5-5.2 mmol/L) despite being on perindopril 10 mg daily and 5 slow-K tablets (600 mg [8 mmol] of potassium chloride per tablet). His aldosterone level was 607 pmol/L ([21.88 ng/dL], RR, 100-950 pmol/L, 3.6-34.25 ng/dL) and direct renin concentration (DRC) was suppressed at 4 mIU/L (RR, 10-50 mIU/L, plasma renin activity 0.5 ng/mL/h [RR 1.2-6 ng/mL/h]). His cortisol level suppressed to 21 nmol/L ([0.76 mcg/dL], RR, <50 nmol/L [<1.8 mcg/dL]) following an overnight 1-mg dexamethasone suppression test. The aldosterone-to-renin ratio was elevated at 152 (RR <71). His medical history included obesity, obstructive sleep apnea, and diabetes. Family history included hypertension. A computed tomography scan of the adrenal glands showed an 8-mm adrenal nodule on the medial aspect of the right adrenal limb (Fig. 1).

## Diagnostic Assessment

For further evaluation including confirmatory testing, the patient’s antihypertensive agents were substituted to verapamil 80 mg 3 times daily and hypokalemia was corrected with potassium supplements (15 slow-K tablets/d). He underwent a seated saline suppression test that failed to suppress aldosterone (1100 pmol/L [39.65 ng/dL] before saline and 777 pmol/L [28.01 ng/dL] after saline [normal <170 pmol/L, 6.13 ng/dL]), confirming the diagnosis of PA. AVS with adrenocorticotropin (ACTH) stimulation was performed (Table 1) and showed adrenal aldosterone to cortisol (A/C) ratios that were less than peripheral A/C ratio despite successful catheterization as evidenced by the adrenal vein to inferior vena cava (IVC) cortisol ratio (selectivity index, SI) of 4 or greater. The AVS was initially interpreted as nonlateralized, and he was medically managed.

**Table 1.** First adrenal vein sampling—under adrenocorticotropin stimulation

	Right AV	IVC	Left AV
Aldosterone	11 280 pmol/L (406.63 ng/dL)	1120.86 pmol/L (40.4 ng/dL)	8360 pmol/L (301.37 ng/dL)
Cortisol	14 800 nmol/L (536.43 mcg/dL)	500.14 nmol/L (18.13 mcg/dL)	11 680 nmol/L (423.34 mcg/dL)
AV cortisol/IVC cortisol ratio (SI)	29.59 (>4)		23.35 (>4)
A/C ratio	0.76	2.24	0.72

Reference range IVC aldosterone: 100 to 950 pmol/L, 3.6 to 34.25 ng/dL; reference range IVC cortisol: 133 to 537 nmol/L, 4.82 to 19.46 mcg/dL. Values in parenthesis are conventional units.

Abbreviations: A/C, aldosterone/cortisol; AV, adrenal vein; AVS, adrenal vein sampling; IVC, inferior vena cava; SI, selectivity index.

Given his family history and relatively young age, he underwent genetic testing for FH-1 gene rearrangement to rule out glucocorticoid remediable aldosteronism, for which he tested negative.

## Treatment

Our patient was commenced on spironolactone 50 mg daily, perindopril 10 mg daily, and amlodipine 10 mg daily for blood pressure control. However, he remained hypertensive and hypokalemic and developed painful gynecomastia from spironolactone, which led to spironolactone being substituted with eplerenone 50 mg twice daily with suboptimal blood pressure control. He also needed potassium replacements to ensure normokalemia—at one point, he needed to take 19 slow-K tablets daily. Despite the use of a mineralocorticoid antagonist and an angiotensin-converting enzyme inhibitor, DRC remained suppressed at 8 mIU/L.

Due to pill burden and medication-related side effects, the previous AVS result was re-reviewed, and it was realized that the patient’s AVS result showed ABAS given low adrenal A/C ratios bilaterally when compared to simultaneous peripheral A/C ratios, despite successful cannulations of both adrenal veins.

**Figure 2.**
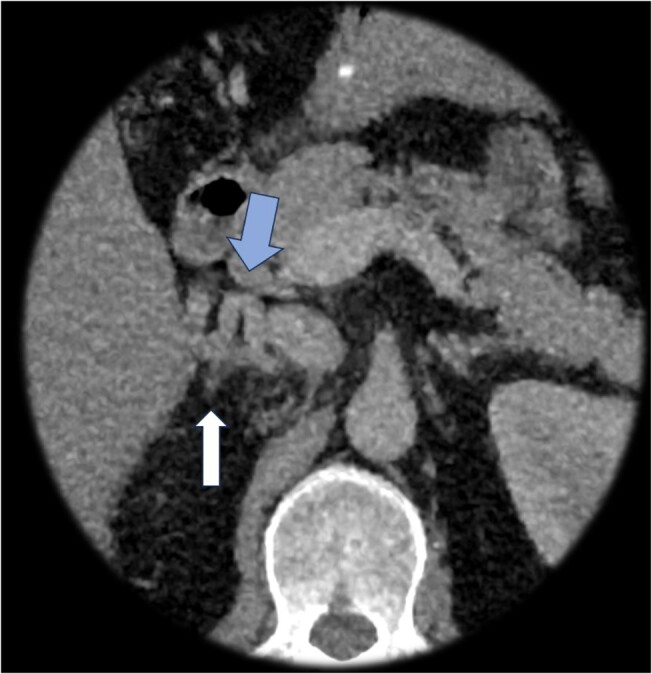
Blue arrow: tortuous periadrenal vein, white arrow: partially imaged right adrenal gland.

**Figure 3.**
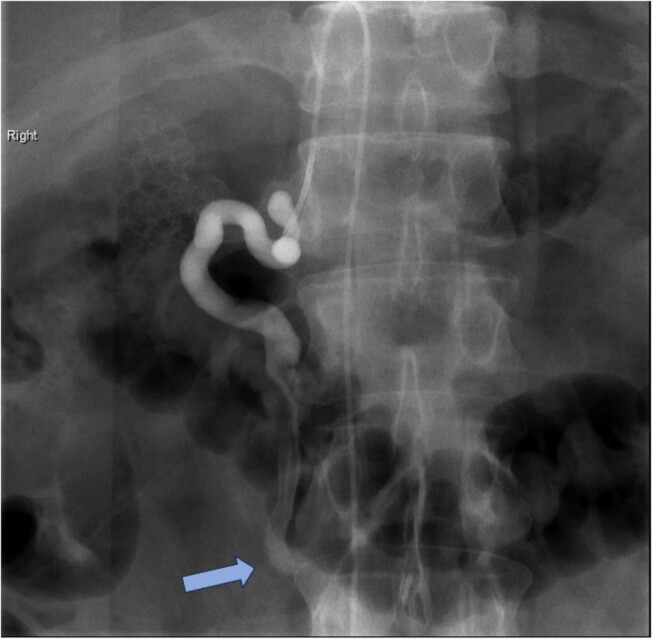
Retrograde flow (blue arrow) via adrenal collateral vein to replaced renal vein and inferior vena cava instead of anterograde flow.

**Figure 4.**
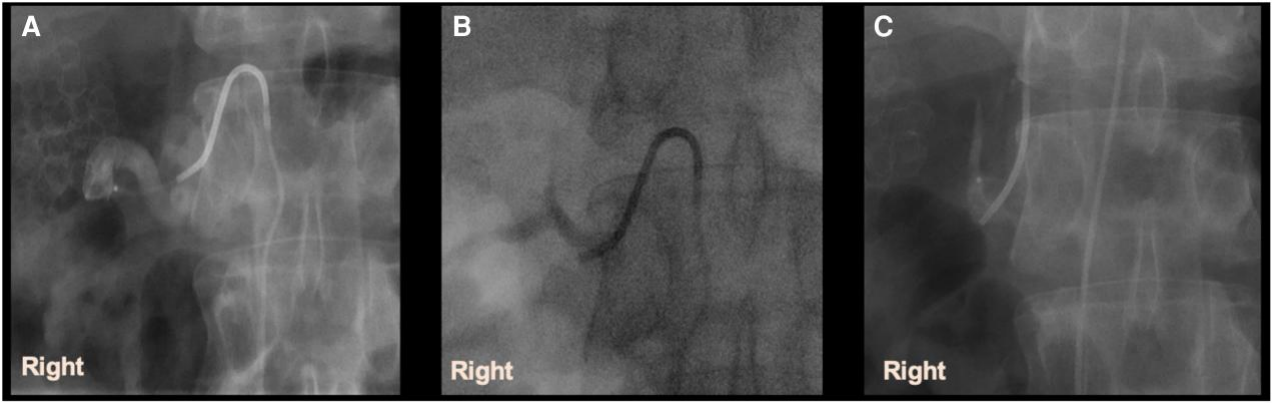
A to C, Selective right adrenal vein sampling using microcatheter and guidewire. “Right” refers to the right adrenal gland.

Review of adrenal vein mapping computed tomography and angiography films revealed an absent right renal vein in our patient, resulting in the recruitment of collaterals for drainage of the right kidney, as well as tortuous periadrenal veins, which made sampling difficult on the right side (Fig. 2). Initial AVS was also complicated by variable directional flow (Fig. 3) from collaterals that replaced the right renal vein. To improve the success of repeat AVS, our radiologist placed the catheter in the hilum of the right adrenal gland and used a microcatheter to sample from multiple collaterals as well (Fig. 4) during the repeat study.

## Outcome and Follow-up

The patient’s repeat stimulated AVS did not confirm ABAS as suggested by the first but lateralized to the right adrenal on more appropriate placement of the catheter with contralateral suppression of the left adrenal gland (Table 2). He then proceeded to laparoscopic right adrenalectomy.

**Table 2.** Second adrenal vein sampling—under adrenocorticotropin stimulation

	Right AV	IVC	Left AV
Aldosterone	52 400 pmol/L (1888.97 ng/dL)	1314.29 pmol/L (47.38 ng/dL)	4720 pmol/L (170.15 ng/dL)
Cortisol	16 112 nmol/L (583.98 mcg/dL)	472.36 nmol/L (17.12 mcg/dL)	23 404 nmol/L (848.28 mcg/dL)
AV cortisol/IVC cortisol ratio (SI)	34.11 (>4)		49.55 (>4)
A/C ratio	3.25	2.78	0.2

Reference range IVC aldosterone: 100 to 950 pmol/L, 3.6 to 34.25 ng/dL; reference range IVC cortisol: 133 to 537 nmol/L, 4.82 to 19.46 mcg/dL. Values in parenthesis are conventional units.

Abbreviations: A/C, aldosterone/cortisol; AV, adrenal vein; AVS, adrenal vein sampling; IVC, inferior vena cava; SI, selectivity index.

**Figure 5.**
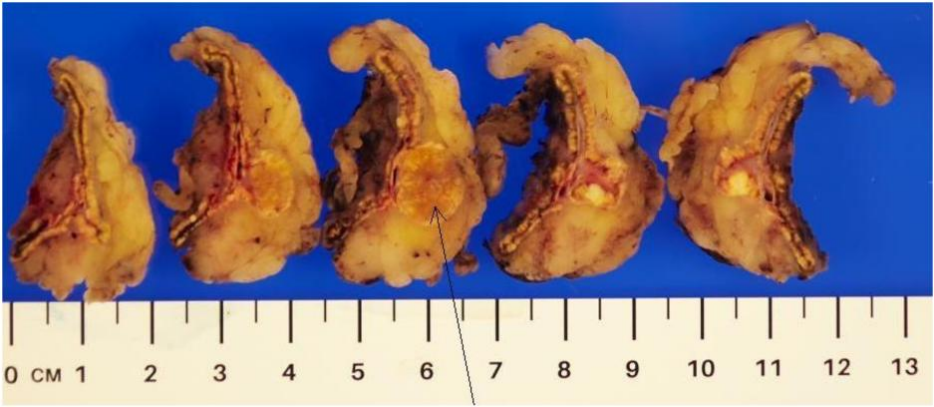
Macroscopic examination of right adrenalectomy specimen. Arrow: solitary adenoma in resected right adrenal gland.

Pathological review of the gross specimen of the right adrenal gland showed an embedded, circumscribed yellow nodule measuring 20 × 10 × 10 mm (Fig. 5).

**Figure 6.**
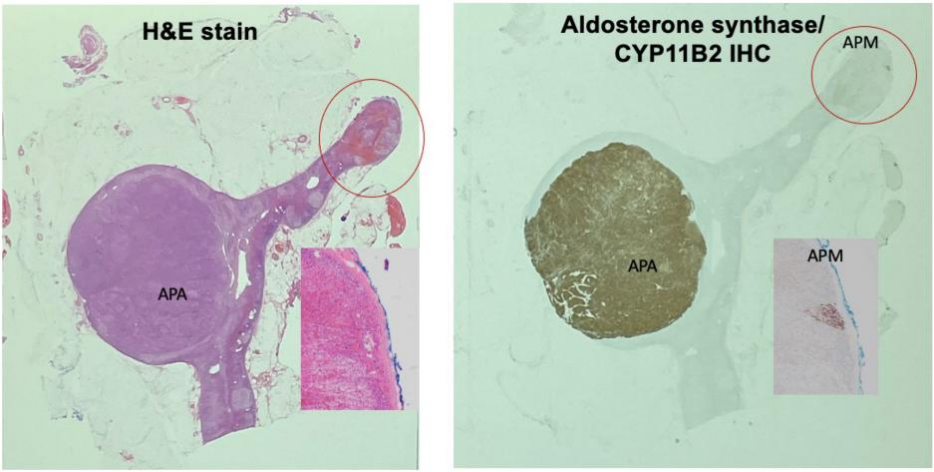
Histopathological analysis of resected adrenal gland with hematoxylin-eosin (H&E) stain and aldosterone synthase/CYP11B2 immunohistochemistry (IHC). APA, aldosterone-producing adenoma; APM, aldosterone-producing micronodule.

On histologic review of the specimen, the circumscribed cortical nodule had clusters of regular bland epithelial cells with adrenal cortical features on hematoxylin-eosin staining. Immunohistochemical evaluation showed positive CYP11B2 (aldosterone synthase) immunostaining in the nodule, in keeping with diagnosis of APA (Fig. 6) and an aldosterone-producing micronodule.

On follow-up, he was normokalemic and normotensive with ramipril 2.5 mg daily to maintain blood pressure. His aldosterone and DRC were 101 pmol/L (3.64 ng/dL) and 9.6 mIU/L, respectively, with an aldosterone-to-renin ratio of 10.

## Discussion

AVS is a critical diagnostic step in subtyping PA. However, it is technically challenging due to difficulties in cannulating the right adrenal vein. To improve the diagnostic yield of AVS, many units perform AVS with ACTH stimulation (5). Additionally, the results obtained from AVS can be imperfect even in bilaterally successful studies and interpreting these results requires expertise and a systematic approach (6). First, successful catheter placement (SI) should be confirmed by an adrenal vein to peripheral vein cortisol ratio. Next, lateralization index (LI) should be calculated by dividing the higher (dominant) adrenal vein A/C ratio with the lower (nondominant) adrenal vein A/C ratio. At our institution, an SI of 4.0 or greater and an LI of 4 or greater with ACTH stimulation indicates successful cannulation of the adrenal vein (2) and unilateral aldosterone excess, respectively. The final step is to calculate the contralateral suppression index by dividing the nondominant adrenal A/C ratio by the peripheral adrenal A/C ratio, with an index of less than 1 predicting surgical outcome and postoperative hyperkalemia in unilateral PA (2).

Rarely, despite correct catheter placement, bilateral adrenal A/C ratios can be lower than peripheral A/C ratios, making these studies uninterpretable. This phenomenon is referred to as apparent bilateral aldosterone suppression (ABAS) or “double down” AVS (6‐8). ABAS was first reported in a case series of patients by Zelinka et al (7) and has subsequently been confirmed by several other groups (8‐11). The prevalence of ABAS has been reported by Wolley et al (8) to be 2.6% in patients who underwent unstimulated AVS. In this series, repeating unstimulated AVS resulted in the resolution of ABAS in 18 of 22 patients, 10 of whom had lateralized disease. In a subsequent paper, the same authors showed the resolution of ABAS following ACTH-stimulated AVS (6). Shibayama et al (9) reported a higher prevalence of 9.5% of ABAS in patients undergoing unstimulated AVS, which was reduced by repeating the sampling with ACTH stimulation (8.7% vs 2.5%). However, ABAS was newly observed in 5 patients following ACTH administration, the explanation of which remains unclear. Since then, there have been additional studies reporting variable prevalence of ABAS (6.6%) in patients undergoing AVS (10, 12). Notably, in many of these studies, preponderance for right-sided disease on repeat sampling has been noted, as in our patient.

Possible explanations for ABAS include sampling error, sampling during quiescent phase of aldosterone secretion as aldosterone secretion from APA can be pulsatile, variant APA venous drainage, and ectopic aldosterone-producing tumor (8‐11, 13). Sampling error could be explained by the difficulties often encountered when cannulating adrenal veins, especially the right adrenal vein, which is small (2-4 mm), has a short trunk, and enters the IVC directly from a posterior-lateral direction (14), resulting in inadvertent super-selective cannulation whereby sampling from the adjacent non-APA adrenal tissue via the non-APA draining tributaries occurs. Placing the catheter more proximally in the right adrenal vein may obviate this problem. Similarly, when sampling the left side, sampling from the common trunk as well as the central adrenal vein may circumvent any ABAS (15). In addition, anatomical variants such as the presence of multiple adrenal veins and drainage into the inferior phrenic vein (on the left side) or into the common trunk of the accessory hepatic vein (on the right side) can also make AVS challenging (16), resulting in sampling from veins that do not drain the APA.

In addition to accidental super-selective sampling of tributary veins and anatomical variants, other explanations for ABAS include fluctuations in aldosterone secretion and rarely ectopic aldosterone-producing tumors (8, 9). Conceivably, if sampling takes place during the quiescent phase of aldosterone production, it may result in a lower adrenal A/C ratio than the peripheral A/C ratio. ACTH stimulation reduces the prevalence of ABAS by reducing sampling during the quiescent phase of aldosterone secretion (11).

DePietro et al (11) evaluated the outcomes of 10 patients with ABAS with repeat ACTH-stimulated AVS and super-selective adrenal vein sampling (SS-AVS). They reported that ACTH-stimulated AVS resolved ABAS in 3 out of 4 patients and SS-AVS resolved ABAS in 6 out of 10 patients (11). SS-AVS collects blood from small adrenal tributary veins, which enables the identification of specific adrenal segments producing aldosterone autonomously. Authors from this study suggested an algorithmic approach to ABAS: 1) repeating AVS with ACTH stimulation if ABAS was encountered during AVS without ACTH stimulation; 2) repeating AVS with ACTH stimulation and SS-AVS if ABAS was encountered during AVS with ACTH stimulation; 3) if ABAS is redemonstrated despite ACTH-stimulated AVS and SS-AVS, considering less common explanations for ABAS (11). Collectively, these studies show that ABAS is often underrecognized and hence missed, leading to delays in diagnosis and definitive management, as in our case.

As our patient had ACTH-stimulated AVS, solely repeating it is less useful. Due to an absent main right renal vein, many collaterals had been recruited to drain the kidney resulting in tortuous periadrenal veins, making right AVS difficult. Additionally, during sampling, retrograde flow via collaterals to the replaced renal vein and IVC was encountered, making blood aspiration difficult. Repeat AVS (with ACTH) was performed and a microcatheter was used to sample from each tributary (super-selective sampling) and hilum, in keeping with DePietro's suggested algorithm in managing ABAS, and was successful in resolving the ABAS.

Despite its challenges and limitations, AVS remains the definitive means to localize PA. ABAS is rare and often is the result of an error in AVS. To avoid misclassifying ABAS as nonlateralized bilateral PA, we recommend that endocrinologists routinely assess A/C ratios individually in both adrenal veins and compare it with peripheral vein A/C ratios as the fifth step in interpreting AVS results (Table 3).

**Table 3.** Steps for interpretation of adrenal vein sampling—our recommendation

Step 1	Confirm successful cannulation by calculating selectivity index and reviewing imaging
Step 2	Correct for dilution (divide adrenal vein aldosterone by the respective cortisol concentration)
Step 3	Determine the aldosterone lateralization ratio
Step 4	Calculate contralateral suppression index
Step 5	Ensure bilateral adrenal aldosterone/cortisol ratios are not lower than peripheral aldosterone/cortisol ratio

When bilateral A/C ratios are lower than peripheral A/C ratios, ABAS should be suspected and strategies to mitigate against ABAS such as repeat sampling with ACTH infusion, performing both SS-AVS and central vein AVS, presampling angiogram (10) and venogram to assess alternate venous drainage, and additional cross-sectional imaging should be considered.

As most patients with ABAS have unilateral PA, it is imperative that AVS be repeated by an experienced radiologist to identify patients who may benefit from adrenalectomy.

## Learning Points

- AVS is challenging, even in the hands of experienced radiologists.
- ABAS is common but remains underrecognized.
- When ABAS is encountered, repeat AVS with strategies to mitigate it including super selective sampling should be considered.
- When interpreting AVS results, clinicians should ensure that adrenal vein A/C ratios are higher than peripheral vein A/C ratio bilaterally.

## Data Availability

Data sharing is not applicable to this article as no data sets were generated or analyzed during the current study.

## Abbreviations

ABAS: apparent bilateral aldosterone suppression
A/C: aldosterone/cortisol
ACTH: adrenocorticotropin
APA: aldosterone-producing adenoma
AV: adrenal vein
AVS: adrenal vein sampling
DRC: direct renin concentration
IVC: inferior vena cava
LI: lateralization index
PA: primary aldosteronism
RR: reference range
SI: selectivity index
SS-AVS: super-selective adrenal vein sampling
